# Social gradients in ADHD by household income and maternal education exposure during early childhood: Findings from birth cohort studies across six countries

**DOI:** 10.1371/journal.pone.0264709

**Published:** 2022-03-16

**Authors:** Nicholas James Spencer, Johnny Ludvigsson, Guannan Bai, Lise Gauvin, Susan A. Clifford, Yara Abu Awad, Jeremy D. Goldhaber-Fiebert, Wolfgang Markham, Åshild Faresjö, Pär Andersson White, Hein Raat, Pauline Jansen, Béatrice Nikiema, Fiona K. Mensah, Jennifer J. McGrath, EPOCH Collaborative Group

**Affiliations:** 1 Division of Health Sciences, Warwick Medical School, University of Warwick, Coventry, United Kingdom; 2 Crown Princess Victoria Children’s Hospital, Region Östergötland, Linköping, Sweden; 3 Division of Pediatrics, Dept of Biomedical and Clinical Sciences, Linköping University, Linköping, Sweden; 4 Department of Public Health, Erasmus MC–University Medical Center Rotterdam, Rotterdam, The Netherlands; 5 Centre de recherche du Centre Hospitalier de l’Université de Montréal, Montréal, Québec, Canada; 6 École de santé publique, Université de Montréal, Montréal, Québec, Canada; 7 Murdoch Children’s Research Institute and Department of Paediatrics, The University of Melbourne, Melbourne, Victoria, Australia; 8 PERFORM Centre, Concordia University, Montreal, Quebec, Canada; 9 Stanford University, Stanford, California, United States of America; 10 Department of Health, Medicine and Caring Science/Inst of Society and Health/Public Health, Linköping University, Linköping, Sweden; 11 Department of Child and Adolescent Psychiatry/Psychology, Erasmus MC–University Medical Center Rotterdam, Rotterdam, the Netherlands; 12 Department of Psychology, Education, and Child Studies, Erasmus MC–University Medical Center Rotterdam, Rotterdam, the Netherlands; 13 Cree Board of Health and Social Services of James Bay, Department of Program Development and Support, Chisasibi, Québec, Canada; The University of Sydney, AUSTRALIA

## Abstract

**Objective:**

This study aimed to examine social gradients in ADHD during late childhood (age 9–11 years) using absolute and relative relationships with socioeconomic status exposure (household income, maternal education) during early childhood (<5 years) in seven cohorts from six industrialised countries (UK, Australia, Canada, The Netherlands, USA, Sweden).

**Methods:**

Secondary analyses were conducted for each birth cohort. Risk ratios, pooled risk estimates, and absolute inequality, measured by the Slope Index of Inequality (SII), were estimated to quantify social gradients in ADHD during late childhood by household income and maternal education measured during early childhood. Estimates were adjusted for child sex, mother age at birth, mother ethnicity, and multiple births.

**Findings:**

All cohorts demonstrated social gradients by household income and maternal education in early childhood, except for maternal education in Quebec. Pooled risk estimates, relating to 44,925 children, yielded expected gradients (income: low 1.83(CI 1.38,2.41), middle 1.42(1.13,1.79), high (reference); maternal education: low 2.13(1.39,3.25), middle 1.42(1.13,1.79)). Estimates of absolute inequality using SII showed that the largest differences in ADHD prevalence between the highest and lowest levels of maternal education were observed in Australia (4% lower) and Sweden (3% lower); for household income, the largest differences were observed in Quebec (6% lower) and Canada (all provinces: 5% lower).

**Conclusion:**

Findings indicate that children in families with high household income or maternal education are less likely to have ADHD at age 9–11. Absolute inequality, in combination with relative inequality, provides a more complete account of the socioeconomic status and ADHD relationship in different high-income countries. While the study design precludes causal inference, the linear relation between early childhood social circumstances and later ADHD suggests a potential role for policies that promote high levels of education, especially among women, and adequate levels of household income over children’s early years in reducing risk of later ADHD.

## Introduction

Attention-Deficit/Hyperactivity Disorder (ADHD) is amongst the most common mental health disorders in childhood. Early studies of the worldwide prevalence of ADHD reported rates between 8–12% [1]. More recent meta-regressions of over 100 studies spanning the globe estimate that the identified prevalence rates hover around 3.4–5.29% [2, 3]. ADHD can be considered as arising from a range of genetic, neurological, and environmental conditions that can act individually or together [4]. ADHD’s heterogenous phenotype is empirically linked to multiple aetiologies including: hereditary polygenic gene variants, neurological factors during brain development, environmental toxin exposure, and early deprivation [5].

ADHD’s association with socioeconomic status (SES) has been reported in a systematic review published in 2016 [6]. Russell et al.’s review identified 42 studies, 35 of which reported a significant association of at least one measure of low SES with ADHD. Across multiple dimensions (i.e., maternal education, paternal education, single parent families, occupation, income), lower SES was robustly associated with ADHD. Children from lower SES families were twice as likely (Odds Ratio 1.85 to 2.21) to have ADHD compared to those from high SES families. Further, Russell and colleagues noted this finding appeared to be universal across continents. Population-based cohort studies, which were published since Russell et al.’s systematic review, have reported an association between household income and/or maternal education in early childhood with ADHD during later childhood in four high-income countries: the UK [7–12], Sweden [13, 14], Denmark [15, 16] and Korea [17]. Reported effect sizes for late childhood ADHD were modest, ranging from an adjusted hazard ratio of 2.09 (95% Confidence Interval (CI) 2.00–2.19) [13] to an unadjusted odds ratio of 1.72 (95% CI 0.94–3.18) [8] for low compared with high household income during early childhood, and an unadjusted odds ratio of 1.45 (95% CI 1.21–1.73) [10] for low compared with high maternal education. Furthermore, a social gradient was evident in eight of these cohort studies [7–9, 13–15, 17]. We are not aware of published studies reporting *absolute* measures of inequality in ADHD. Indeed, few studies compare the association of ADHD with SES using compatible measures in more than one country; one cross-sectional study reported on the association in five European countries [18] and one study compared results from a UK and Brazilian birth cohort [10].

The measurement of ADHD is fundamental when comparing prevalence rates and their determinants across countries. Diagnostic definitions have evolved over time: ‘minimal brain dysfunction’ and ‘organic brain dysfunction’ morphed into ‘hyperkinetic disorder’, ‘attention deficit disorder’, and ‘attention deficit hyperactivity disorder’. ICD and DSM are the two international medical classification systems used globally. The ICD-10 definition of Hyperkinetic Disorders with disturbance of activity and attention (F90.0) [19] differs from the DSM-5 definition of Attention-deficit/Hyperactivity Disorder (314.0X) [20]; ICD-10 yields a lower prevalence rate because of its more stringent criteria requiring symptoms for inattention, hyperactivity, and impulsivity [21]. These differences in definition likely explain differences in prevalence rates between Europe and North America as the ICD-10 definition is more commonly used in Europe and the DSM-5 is more commonly used in the US and the rest of the world. The more recent ICD-11 introduces emerging convergence toward a consensus definition with criteria closely aligned with the DSM-5 [22]. Other diagnostic differences across countries are also relevant, including which health professionals are licensed to diagnose ADHD (e.g., primary care clinicians, pediatricians, general family physicians, psychiatrists, clinical psychologists, social workers, other health professionals), the clinical assessment method used, and the rigor of applying diagnostic criteria. Of particular relevance to this study, ADHD diagnosis may be limited by income, insurance, access to a diagnostic professional, or parental engagement for seeking assessment.

The Elucidating Pathways Of Child Health inequalities (EPOCH) study draws on data from seven birth cohort studies from six countries to explore the pathways from early SES exposure to child health outcomes at age 9–11 years. The EPOCH study, funded by the Canadian Institutes of Health Research (CIHR), was conceived and planned by researchers in the International Network for Research on Inequalities in Child Health (INRICH) [www.inrichnetwork.org]. Researchers reviewed and considered large, population-based birth cohorts from their respective countries that had comparable data aligned with the UNICEF child well-being outcomes. We acknowledge potential bias in selection of participating countries due to data accessibility; however, all included countries are high income with contrasting social policies and distributed along the gradient of country-level measures of income inequality. In addition, the birth cohort enrollments were largely conducted during the same time period (i.e., age 0–1 recruitment years: 1997–2004), with the exception of the US cohort (i.e., recruitment years: 1988–1996). The impact of low socioeconomic status in early childhood on adult health has been extensively studied [23], but less attention has been given to its impact across childhood and adolescence [24]. The EPOCH study aims to address this gap in the literature. This project within the EPOCH study explores pathways to ADHD. The paper aims to examine the relationship of household income and maternal education as separate measures of SES exposure during early childhood to ADHD during late childhood in the different country settings represented by the participating cohorts.

## Methods

### Data sources

Data were extracted from seven birth cohorts participating in the EPOCH study: UK Millennium Cohort Study (MCS); Alla Barn i Sydöstra Sverige (All Babies in Southeast Sweden, ABIS); Quebec Longitudinal Study of Child Development (QLSCD); Longitudinal Study of Australian Children B cohort (LSAC B); Generation R, Rotterdam, The Netherlands (GenR); National Longitudinal Study of Children and Youth, Canada (NLSCY); and National Longitudinal Study of Youth, USA (US NLSY). Concordia University Human Research Ethics Committee certified the ethical acceptability for EPOCH’s secondary data use (#2011028). All original birth cohorts complied with the ethical standards of their relevant institutional and/or national committees and with the Helsinki Declaration of 1964, and its later amendments. As part of the original cohort methodology, information summarizing what participation would involve was provided in both oral and written form; explicit informed written consent was obtained for all participants (parents, guardians). Cohort profiles and cohort technical report references are shown in Table 1. All cohorts enrolled population-based samples of children at birth or within the first 2 years of life. The US cohort was based on the 1996 Child Supplement and included children who were born to female participants of the nationally representative 1979 National Longitudinal Survey of Youth (NLSY79). The Southeast Sweden cohort, based on comprehensive medical records and linked to administrative databases, had the lowest attrition rate (3.9%) whereas attrition rates for the remaining cohorts varied between 24.2% (Rotterdam, The Netherlands) and 39.1% (Canada, all provinces). Weights and/or imputation accounting for differential attrition and non-response were applied in all cohorts except Southeast Sweden.

**Table 1:** Cohort profiles.

Cohort Country/Region Baseline Year	Age at Baseline & Cohort Follow-ups^a^	Sampling Methodology	Sample Size (N) Baseline & 10/11 yrs Attrition Rate^b^ (%)	Weighting & Imputation for Attrition
MCS [25] UK 2000	Baseline (Sweep 1): 9 mos Sweep 2: 3 yrs Sweep 3: 5 yrs Sweep 4: 7 yrs Sweep 5: 10/11 yrs	• “All children born between September 1, 2000 and August 31, 2001 (for England and Wales), and between November 24, 2000 and January11, 2002 (for Scotland and Northern Ireland), alive and living in the UK at age 9 months, and eligible to receive child benefit at that age” • Eligibility based on government child benefit records (i.e., nearly universal coverage); asylum seekers not eligible • Subgroups intentionally oversampled (living in disadvantaged areas, ethnic minorities)	Baseline: 18,552 10/11 yrs (Sweep 5): 13,354 Attrition: 28.4%	• Weights applied • Imputation for differential attrition
ABIS [26, 27] Southeast Sweden 1997 to 1999	Baseline (Sweep 1): Birth Sweep 2: 1 yr Sweep 3: 2.5 yrs Sweep 4: 5 yrs Sweep 5: 8 yrs Sweep 6: 10–12 yrs	• All children born October 1, 1997 to September 30, 1999 in a defined region in southeast of Sweden were invited	Baseline: 17,055 10/12 yrs (Sweep 6): 16,365 Attrition: 4.0%	• No weights applied • No imputation
QLSCD [28] Quebec, Canada 1997	Baseline (Wave 1): 6 mos Wave 2: 1.5 yrs Wave 3: 2.5 yrs Wave 4: 3.5 yrs Wave 5: 4 yrs Wave 6: 5. yrs Wave 7: 6 yrs Wave 8: 7 yrs Wave 9: 8 yrs Wave 10: 10 yrs	• All singleton live births, born in 1997 from mothers living in Quebec, except in First Nation’s territories • Excluded very premature or post term birth, and when sex or gestational age were unknown	Baseline: 2,120 10 yrs (Wave 10): 1,334 Attrition: 37%	• Weights applied (i. Transversal to adjust for participation for a given wave; ii. Longitudinal to account for differential attrition) • No imputation
LSAC B [29] Australia 2004	Baseline (Wave 1): Birth-1yr Wave 2: 2–3 yrs Wave 3: 4–5 yrs Wave 4: 6–7 yrs Wave 5: 8–9 yrs Wave 6: 10–11 yrs	• National sample using two-stage random sampling design: (1) random selection of 10% of postcodes, stratified by state and urban/rural locations), (2) random selection of in-age children within those postcodes from Medicare (universal healthcare) database • Excluded very remote postcodes and postcodes with <20 children (n = 874 postcodes, 3.2% of population).	Baseline: 5,107 10–11 yrs (Wave 6): 3,764 Attrition: 26.1%	• Weighted back to the reference population • Weights adjusted for non-response at each wave
GenR [30, 31] Rotterdam, The Netherlands 2002 to 2006	Baseline (Wave 1): Birth-4yrs (”Preschool Period”: 2 mos, 6 mos, 1 yr, 1.5 yrs, 2 yrs, 3 yrs, 4 yrs) Wave 2: 5–6 yrs Wave 3: 9–10 yrs	• Pregnant women who expected to deliver between April 2002 and January 2006, living in Rotterdam, who visited a midwife or obstetrician were eligible for participation and contacted by GenR staff.	Baseline: 9,749 9–10 yrs (Wave 3): 7,393 Attrition: 24.2%	• Weights applied to account for differential attrition • No imputation
US NLSY [32] USA 1988 to 1996	Baseline: Birth Round 2: 2 yrs Round 3: 4 yrs Round 4: 6 yrs Round 5: 8 yrs Round 6: 10 yrs	• Original NLSY79 cohort was cross-sectional, population representative sample born between January 1958 and December 1964; subsamples intentionally included Hispanic or Latino, black, economically disadvantaged nonblack/non-Hispanic, and military personnel • NLSY79 Child and Young Adult cohort follows offspring born to female respondents of the original NLSY79 cohort. Analytical sample for present study was limited to children born between 1988 to 1996.	Baseline: 3,657 10 yrs (Round 6): 2,976 Attrition: 18.6%	• Weights applied to account for differential attrition and to weight back to the population • No imputation
NLSCY [33] Canada 2000 to 2004	Baseline: Birth– 11 mos Cycle 2: 2 yrs Cycle 3: 4 yrs Cycle 4: 6 yrs Cycle 5: 8 yrs Cycle 6: 10 yrs	• Sampling was conducted in collaboration with Canada’s Labour Force Survey and National Population Health Survey • Sampling was stratified by province to select a representative sample of children in Canada	Baseline: 2,227 10 yrs (Cycle 6): 1,356 Attrition: 39.1%	• Weights applied to account for differential attrition and to weight back to the population • No imputation

Notes

^a^Follow-ups specific to the timeframe of present study (birth to age 10); several cohorts are ongoing. Follow-up terminology preserved (e.g., waves, sweeps); when no term given, “wave” was used for clarity.

^b^Sample size at age 10 yrs was based on complete cases with all variables observed to yield conservative attrition rate estimate.

### ADHD outcome variable

Attention Deficit Hyperactivity Disorder (ADHD) was measured in late childhood. Table 2 shows the measurement specifications for each cohort. All cohorts were based on DSM-IV or ICD-10 criteria. ADHD diagnosis was based on parental report of medical diagnosis or diagnostic criteria for all cohorts, except ABIS, which was derived from Swedish medical records. The Dutch cohort (Netherlands, GenR) used a DSM-oriented symptom scale with a predefined clinical cut-point (98^th^ percentile) and the timeframe was limited to past 6 months. The US cohort (US NLSY) recorded ‘limiting’ hyperkinesis/hyperactivity/attention deficit disorder, whereas all other cohorts recorded ‘ADHD ever diagnosed’.

**Table 2:** ADHD measurement harmonization across cohorts.

Cohort	ADHD Diagnosis			Measurement			Specification/Details
	Classification System	Informant	Format	Years ADHD assessed	Child Age	Time Span	
MCS	ICD-10	Parent (96% mother)	Interview	2010–2011	10–11 yrs	Ever	“Has a doctor or health professional ever told you that [*child name*] had any of the following problems? Attention Deficit Hyperactivity Disorder (ADHD)”
UK					*M* = 11.2yrs		
ABIS	ICD-10	Medical Record	Code	2007–2009	10–11 yrs	Ever	ICD-10 ADHD diagnosis (coded by ICD-10; F90.0) from linked medical records in the National Patient Register
Sweden					*M* = 11yrs		
QLSCD	DSM-IV	Parent (98% mother)	Interview	2007–2008	10 yrs	Ever	“Has a doctor or health professional ever told you that [*child name*] had any of the following problems? Attention deficit disorder with or without hyperactivity”
Quebec					*M* = 10.1yrs		
LSAC B	DSM-IV	Parent (96% mother)	Questionnaire	2014–2015	10–11 yrs	Ever	“Does [*child name*] have any of these ongoing conditions? (Ongoing conditions’ exist for some period of time (weeks, months or years) or re-occur regularly. They do not have to be diagnosed by a doctor.) ADD/ADHD”
Australia					*M* = 10.9 yrs		
GenR	DSM-IV	Parent (100% mother)	Questionnaire	2011–2014	9–10 yrs	Past 6 mos	DSM-Oriented Scales for Attention Deficit/Hyperactivity Problems (7 questions from Child Behavior Checklist^a^). Clinical cut-point defined as 98^th^ percentile (raw score cut-point unique to boys and girls)
Rotterdam, The Netherlands					*M* = 9.7 yrs		
US NLSY	DSM-IV	Parent (100% mother)	Interview	1998–2008	9–12 yrs	Ever	“Does [*child name*] have any physical, emotional, or mental condition that limits or prevents his/her ability to…? What condition–hyperkineses/hyperactivity/attention deficit disorder”
USA					*M* = 10.5 yrs		
NLSCY	DSM-IV	Parent or Person Most Knowledgeable (88% mother)	Interview	2004–2005	9–12 yrs	Ever	“Has a health professional diagnosed any of the following long-term conditions for [*child name*]? (Long-term conditions refer to conditions that have lasted or are expected to last 6 months or more and have been diagnosed by a health professional.) Attention deficit disorder (with or without hyperactivity)”
Canada					*M* = 10.1 yrs		

Note.

^a^Child Behavior Checklist 98^th^ percentile cut-point is not a diagnostic equivalent, but suggests consideration by a licensed professional. (Achenbach TM. 1991. Manual for Child Behavior Checklist/ 4–18 and 1991 Profile. Burlington: University of Vermont.)

### Main independent variables of interest

Household income and maternal education are two of the most common measures of SES in observational studies [34]. Household income and maternal education, measured in the first five years of life, were available in all cohorts allowing harmonisation of these key variables. Household income tertiles at birth or within early life with ranges and means in local currency and $Purchasing Power Parity 2000 ($PPP) [35] were obtained for each cohort. Table 3 shows differences in income data collection. Four cohorts collected household income net of tax and transfers; the three other cohorts collected gross income. Three cohorts reported equalized income that was derived by dividing household income by a factor calculated based on the number of household members as follows: the first adult member of the household is given an equivalence value of 1.0, the second adult 0.5 and each subsequent person aged 14 and over 0.5, each child aged under 14 adds a value of 0.3 [36]. $PPP ranges for the low income groups vary widely; low income households in the Rotterdam, The Netherlands cohort had the highest purchasing power, while low income households in the UK cohort had the lowest. Maternal education at child’s birth or within first year of life was harmonized to high, middle, and low using the International Standard Classification of Education (ISCED): low education = ISCED I-II; middle education = ISCED III-IV; high education = ISCED V-VII [37].

**Table 3:** Income data collection and ranges by cohort.

Cohort	Annual Income (Gross or Net)	Early Childhood Household Income Assessment Age	Equivalised (Yes or No)	Annual Income Range & Mean by Local Currency & $PPP		
				High Income	Middle Income	Low Income
MCS	Net	9 mos	Yes	Range: >£17,248 (>$PPP 24,493)	Range: £9,131-£17,243 ($PPP 12,984–24,492)	Range: <£9,136 ($PPP <12,979)
UK			(OECD)			
				Mean: £28,075 ($PPP 39,879)		Mean: £6,193 ($PPP 8,798)
					Mean: £13,598 ($PPP 19,313)	
ABIS	Net	1–3 yrs	No	Range: >315,843 SEK ($PPP >34,466)	Range: 263,827–315,842 SEK ($PPP 28,787–34,466)	Range: <263,827 SEK ($PPP <28,787)
Sweden						
				Mean: 419,952 SEK ($PPP 45,864)		Mean: 206,648 SEK ($PPP 22,568)
					Mean: 289,744 SEK ($PPP 31,616)	
QLSCD	Gross	Before birth (-1 yr, before maternity leave)	Yes	Mean: CAD 41,962 ($PPP 34,171)	Mean: CAD 11,432 ($PPP 9,310)	Mean: CAD 9,068 ($PPP 7,384)
Quebec, Canada						
LSAC B	Gross	Birth-1 yrs	No	Range: >AUD 68,432 ($PPP >50,097)	Range: AUD 42,706–68,380 ($PPP 31,253–50,058)	Range: <AUD 42,692 ($PPP <31,253)
Australia						
				Mean: AUD 109,445 ($PPP 80,120)	Mean: AUD 54,865 ($PPP 40,165)	Mean: AUD 29,354 ($PPP 21,489)
GenR	Net	5 yrs	Yes	Range: >€48,000 ($PPP >53,928)	Range: €28,800–48,000 ($PPP 32,364–53,928)	Range: <€28,800 ($PPP <32,364)
Rotterdam, The Netherlands						
US NLSY^a^	Net	0–2 yrs	No	Range:	Range:	Range:
USA				1988: ≥34,701 USD	1988: 16,300–34,700 USD	1988 ≤16,299 USD
				1990: ≥40,701 USD	1990: 21,970–40,700 USD	1990 ≤21,969 USD
				1992: ≥49,601 USD	1992: 25,790–49,600 USD	1992 ≤25,789 USD
				1994: ≥59,601 USD	1994: 31,400–59,600 USD	1994 ≤31,399 USD
				1996: ≥61,501 USD	1996: 30,250–61,500 USD	1996 ≤30,249 USD
NLSCY	Gross	0–11 mos	No	Range: CAD >50,000 ($PPP 44,200)	Range: CAD 30,000–50,000 ($PPP 26,500–44,200)	Range: CAD <30,000 ($PPP <26,500)
Canada						

Note. $PPP purchasing power parity.

^a^US NLSY baseline (ages 0–2 yrs) ranged from years 1988–1998; tertile range reported for each year.

### Baseline confounding variables

Cohort surveys defined ethnicity using ‘Majority / Minority’ or ‘Born inside country / Born outside country’ designations; Mother Ethnicity was dichotomized using these designations. Aboriginal (Australia) and First Nation (Quebec) mothers were classified as ‘Born in country’ in these cohorts. Additional confounding variables included maternal age at child’s birth, child sex, and multiple births. The Quebec cohort did not include multiple births as it had singleton births only.

### Statistical analyses

We estimated unweighted frequencies for all variables. We estimated unadjusted and adjusted Risk Ratios (RRs) using a generalized linear model with a log link and robust variance estimation (see S1 Appendix for details of data analysis in each cohort) [38]. The level of significance was set at p < .05 (two-tailed) for all analyses. Potential mediators identified in previously published literature (smoking in pregnancy [7], birth weight [10], lone parenthood [39], breast feeding [40], low parental involvement [7], maternal mental health [12]), which are potentially on the pathway between early childhood SES and ADHD in late childhood were excluded from the regression models to avoid overcontrolling for the effect of SES. In cohorts with loss to follow-up, either censoring and/or sample weights were used to adjust for nonresponse and/or make the sample comparable to a reference population. Only the Southeast Sweden cohort did not use sample weights because the cohort did not experience differential attrition due to the use of nationwide health records containing ADHD diagnosis information for all children.

After Risk Ratios were estimated, they were pooled using the Meta for package in R [41]. We estimated the I^2^, which is the percentage heterogeneity in RRs among studies, relative to the total amount of variance. The Slope Index of Inequality (SII) was estimated for each population. SII estimates the absolute advantage of higher income or education, interpreted as the absolute difference in percentage of ADHD from the highest to the lowest level in the social hierarchy. SII is a regression-based index that accounts for the socioeconomic distribution of the population, excluding the size of socioeconomic groups as a source of variability in estimating the magnitude of inequalities in health [42]. Differences in ADHD prevalence between the least and the most advantaged groups were estimated for each cohort by extrapolation from the weighted prevalence estimates for each of the income tertiles and the three levels of maternal education. The SIIs were weighted to adjust for differential loss to follow-up in GenR and to weight back to the population in the NLSCY, MCS, LSAC, QLSCD and USNLSY cohorts. If given a causal interpretation, the SII estimates how much ADHD prevalence would be reduced if income or maternal education were improved in each population. Compared to relative risks, absolute estimates of risk have a different public health utility as they reflect the magnitude of a health outcome within a population; thus, instead of comparing risks among groups, absolute risks convey what percentage of the population is affected. On the other hand, relative risks are multiplicative; they show how much more disadvantaged groups are affected compared to wealthier counterparts. Relative risks convey relative inequality, and therefore, are useful when considering issues of social justice.

## Results

Samples for all cohorts were drawn from whole populations and were broadly representative of the target populations (Table 1). The design of the US cohort resulted in no children born to mothers under the age of 24 years being enrolled, thus excluding at least 5% of the child population [43]. Numbers of children with complete data on exposures in early childhood and ADHD in late childhood are shown in Table 4. Pooled estimates relate to a total of 44,925 children in late childhood. Cohort sample characteristics by unweighted frequencies are shown in Table 4. Prevalence of ADHD ranged from 1.3% in the UK to 7.6% in Quebec. Levels of maternal education at baseline varied widely by cohort: Quebec and UK cohorts have the largest proportion of mothers with low education (28.6% & 20.8%, respectively) and Sweden and Australia, the smallest (8.4% & 9.0% respectively); the proportion of mothers with high education was larger in Australia (47.8%) and Canada (all provinces, 42%), than in the remaining cohorts. Proportion of mothers from ethnic minority groups or born outside the cohort country was lowest in Southeast Sweden (6.5%) and highest in the US cohort (43.9%); the US cohort (US NLSY) purposely oversampled minority groups (n.b., sample weights applied to inferential statistics adjusted for oversampling). Proportion of mothers below 20 years of age at their child’s birth in the Canada cohort (23%) was high and an outlier compared with all other cohorts with proportions less than 10%.

**Table 4:** Sample characteristics by cohort (unweighted frequencies).

Variables TOTAL N = 44,925		MCS	ABIS	QLSCD	LSAC B	GenR	US NLSY	NLSCY
		UK	Sweden	Quebec	Australia	Rotterdam	USA	Canada
		(*N* = 13354)	(*N* = 16365)^a^	(*N* = 1334)	(*N* = 3759)^a^	(*N* = 5100)^a^	(*N* = 3657)^a^	(*N* = 1356)
Child Sex	Male	6730 (50.4%)	8485 (51.8)	688 (51.6%)	1928 (51.3%)	2557 (50.1%)	1881 (51.4)	687 (50.7%)
	Female	6624 (49.6%)	7880 (48.2)	646 (48.4%)	1831 (48.7%)	2543 (49.9%)	1776 (48.6)	669 (49.3%)
Mother Age at Child Birth^b^	<20 yrs	925 (6.9%)	226 (1.4%)	37 (2.8%)	74 (2.0%)	65 (1.3%)	0	310 (23%)
	20–29	5809 (43.5%)	8638 (52.8%)	663 (49.7%)	1337 (35.6%)	1712 (33.6%)	1767 (48.3%)	451 (33%)
	30–39	5849 (43.8%)	6817 (41.7%)	603 (45.2%)	2192 (58.3%)	3200 (62.7%)	1887 (51.6%)	435 (32%)
	40+	293 (2.2%)	293 (1.8%)	30 (2.2%)	153 (4.1%)	123 (2.4%)	0	129 (10%)
	Missing	478 (3.6%)	391 (2.4%)	1 (0.1%)	3 (0.1%)	0	3 (0.1%)	16 (1%)
Mother Ethnicity	Terminology	Majority/Minority	Majority /Minority	Born in country/Born outside	Born in country/Born outside	Majority/Minority	Majority/Minority	Born in country/Born outside
Ethnic Majority/Born in country		10919 (81.8%)	14960 (91.4%)	1161 (87.0%)	2453 (65.3%)	3035 (59.5%)	2050 (56.1%)	1232 (90.9%)
Ethnic Minority/Born outside country		1958 (14.7%)	1062 (6.5%)	171 (12.8%)	1297 (34.5%)	2065 (40.5%)	1607 (43.9%)	123 (9.1%)
Missing		477 (3.6%)	343 (2.1%)	2 (0.1%)	9 (0.2%)	0	0	1 (0.1%)
Multiple Births	Yes	343 (2.6%)	380 (2.3%)	0	132 (3.5%)	104 (2.0%)	91 (2.5%)	36 (2.7%)
	No	12534 (93.9%)	15985 (97.7%)	1334 (100%)	3626 (96.5%)	4996 (98.0%)	3566 (97.5%)	1283 (94.6%)
	Missing	477 (3.6%)	0	0	1 (0.0%)	0	0	37 (2.7%)
Outcome: ADHD by Late Childhood	Yes	175 (1.3%)	288 (1.8%)	102 (7.6%)	133 (3.5%)	120 (2.4%)	107 (2.9%)	66 (4.9%)
	No	13009 (97.4%)	16077 (98.2%)	1231 (92.3%)	3563 (94.8%)	3594 (70.5%)	2982 (81.5%)	1240 (91.4%)
	Missing	170 (1.3%)	0	1 (0.1%)	63 (1.7%)	1386 (27.2%)	568 (15.5%)	50 (3.7%)
Exposure: Income in Early Childhood	High	3936 (29.5%)	5417 (33.1%)	463 (34.7%)	1384 (36.8%)	1794 (35.2%)	951 (26.0%)	544 (40.1%)
	Middle	4162 (31.2%)	5417 (33.1%)	434 (32.5%)	1327 (35.3%)	1772 (34.7%)	932 (25.5%)	436 (32.2%)
	Low	4651 (34.8%)	5418 (33.1%)	381 (28.6%)	1048 (27.9%)	1534 (30.1%)	1093 (29.9%)	376 (27.7%)
	Missing	605 (4.5%)	113 (0.7%)	56 (4.2%)	0	0	681 (18.6%)	0
Exposure: Maternal Education at Baseline	High	4176 (31.3%)	5068 (31.0%)	394 (29.5%)	1796 (47.8%)	1464 (28.7%)	1073 (29.3%)	567 (41.8%)
	Middle	5544 (41.5%)	9525 (58.2%)	557 (41.8%)	1624 (43.2%)	2700 (52.9%)	1922 (52.6%)	568 (41.9%)
	Low	2782 (20.8%)	1379 (8.4%)	382 (28.6%)	337 (9.0%)	936 (18.4%)	657 (18.0%)	187 (13.8%)
	Missing	852 (6.4%)	393 (2.4%)	1 (0.1%)	2 (0.1%)	0	5 (0.1%)	34 (2.5%)

Note.

^a^Sample size numbers may differ from *N* reported in Table 1 due to missing data for SES exposures in early childhood or ADHD in late childhood, or cohort attrition.

^b^Sample size for mother age at child birth differs for NLSCY due to different age categories.

Unadjusted Relative Ratios (i.e., bivariate risk ratios; RRs) of ADHD by income tertiles, the three categories of maternal education, and confounding variables (i.e., child sex, mother age at birth, mother ethnicity, multiple births) are shown in S1 Table. Adjusted RRs, adjusting for all confounding variables, are shown in Table 5. For maternal education categories, social gradients were present in all cohorts, except Quebec. Additionally, confidence intervals around both estimates in the Canada (all provinces) and US cohorts crossed unity (see Table 5). For household income tertiles, adjusted RRs for ADHD by household income tertiles showed a tendency towards social gradients in all cohorts, except for the Quebec cohort, where the risk was higher for middle than low income; confidence intervals for both estimates crossed unity in the Australian, US, and Canada (all provinces) cohorts.

**Table 5:** Risk ratios for ADHD in late childhood by income and maternal education at baseline using adjusted multivariate regression.

	MCS	ABIS	QLSCD	LSAC B	GenR	US NLSY	NLSCY
	UK	Sweden	Quebec	Australia	Rotterdam	USA	Canada
Household Income							
High (Reference)	Reference	Reference	Reference	Reference	Reference	Reference	Reference
Middle	1.53 (0.84, 2.80)	1.13 (0.83, 1.54)	1.79 (1.05, 3.05)	0.99 (0.63, 1.57)	1.64 (1.01, 2.56)	1.34 (0.76, 2.34)	0.46 (0.19, 1.07)
Low	4.20 (2.30, 7.67)	1.59 (1.18, 2.14)	1.63 (0.90, 2.94)	1.24 (0.78, 1.97)	2.19 (1.32, 3.64)	1.46 (0.81, 2.65)	1.94 (0.96, 3.93)
Maternal Education							
High (Reference)	Reference	Reference	Reference	Reference	Reference	Reference	Reference
Middle	1.44 (0.85, 2.44)	1.85 (1.33, 2.56)	0.83 (0.48, 1.42)	1.32 (0.86, 2.02)	1.84 (1.43, 2.37)	1.10 (0.68, 1.78)	1.21 (0.46, 3.15)
Low	3.61 (2.05, 6.33)	3.98 (2.65, 5.98)	0.88 (0.59, 1.80)	2.75 (1.60, 4.58)	2.04 (1.46, 2.37)	1.27 (0.64, 2.51)	1.70 (0.50, 5.85)

Note: Risk ratios adjusted for child sex, mother age at birth, mother ethnicity, and multiple births for all cohorts, except QLSCD.

**Fig 1:**
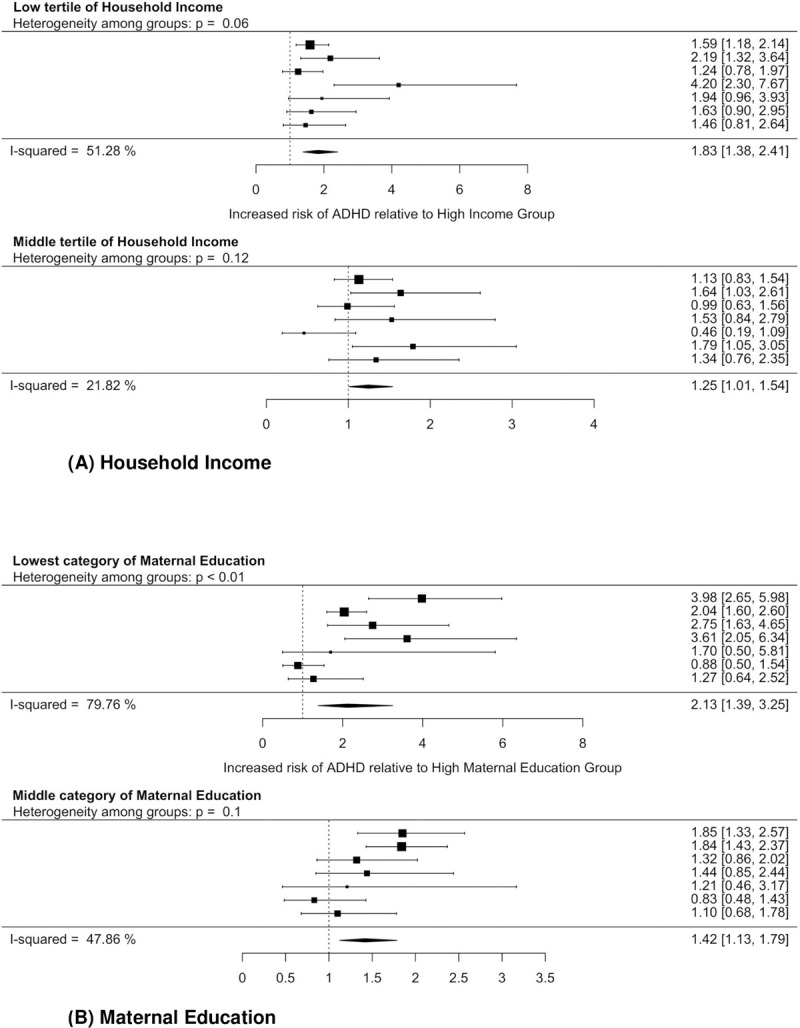
Forest plots of ADHD prevalence by income and education. (A) Household Income. (B) Maternal Education.

Pooled estimates were calculated for household income (low: 1.83, 95% CI = 1.38, 2.41; middle: 1.42, 95% CI = 1.13, 1.79) and maternal education (low: 2.13, 95% CI = 1.39, 3.25; middle: 1.42, 95% CI = 1.13, 1.79). Heterogeneity ranged from moderate to high (I^2^ middle income 47.9%, middle education 47.9%, low income 51.3%, low education 79.8%). (Forest plots are shown in Fig 1).

**Fig 2:**
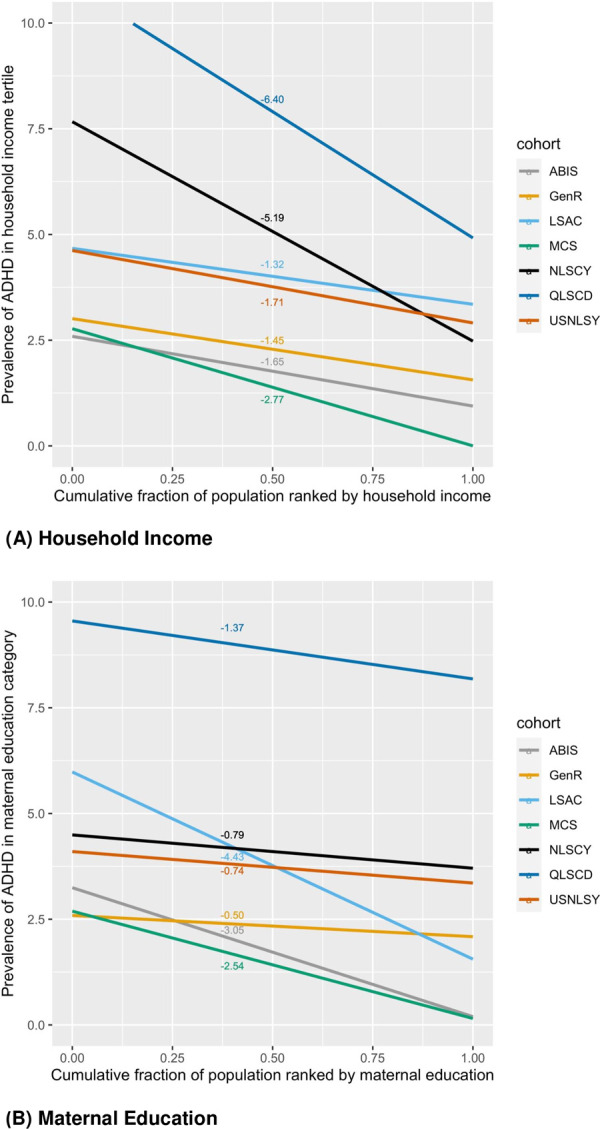
Slope index of inequalities (SIIs) plots by income and education. (A) Household Income. (B) Maternal Education.

Absolute inequality in ADHD during late childhood across the cohort populations confirms the advantage for children in high income households or those with more highly educated mothers in most cohorts. If we were to assign a causal interpretation to these estimates, the largest potential reduction in ADHD prevalence due to increased maternal education levels would be observed in Australia and Sweden (reduction in ADHD: 4% and 3%, respectively); while improvement in household income would lead to the largest reductions in Quebec and Canada (reduction in ADHD: 6% and 5%, respectively). These findings may seem to contradict the impression given by Relative Risks that the most severe inequalities by income are found in the UK and The Netherlands (RR of low vs high income: 4.2 and 2.19, respectively). However, while the poorest in these countries are at higher risk of ADHD compared to the wealthiest, the lower prevalence of ADHD means lower absolute risk at the population level. On the other hand, income changes would result in larger reductions in Canada where the prevalence of ADHD is higher. It should also be noted that while Relative Risks were adjusted for confounders (child sex, mother age at birth, mother ethnicity, multiple births), the SIIs were not. (SIIs plots are shown in Fig 2).

## Discussion

The objective of the EPOCH study, of which this paper is a part, is to examine the pathways from SES around birth and in the first 5 years of life and a range of child health outcomes at 9–11 years of age in different high income country settings. An essential step in studying these pathways is to demonstrate social gradients in these outcomes in the participating cohorts Although the study design limits causal inference, the finding of social gradients and the linear relationship by SES measures at baseline with ADHD during late childhood lends support to a possible causal role. To our knowledge, this is the first published paper examining social gradients in ADHD in later childhood, using relative and absolute measures of inequality, by maternal education and household income in early childhood based on comparable data in cohort studies from different industrialised countries. The findings indicate that high SES exposure during early childhood, measured as maternal education or household income, is associated with lower risk of ADHD later at age 9–11. Pooled estimates showed that low maternal education at baseline was associated with a 113% higher risk of ADHD during late childhood, relative to high maternal education; middle maternal education was associated with a 42% higher risk. The equivalent risk increase for low household income was 83% and for middle household income was 42%, relative to high household income. Absolute inequality in ADHD in late childhood across the cohort populations confirms the advantage for children with more highly educated mothers or in high income households in most cohorts. Absolute inequality differs from relative inequality as it reflects the prevalence of the outcome, while accounting for the proportion of each level of SES in the population. The SII provides an estimate of the difference in ADHD prevalence between the highest and lowest levels of household income and maternal education in the cohort populations. Absolute inequality in ADHD has not previously been reported and, in combination with relative inequality, provides a more complete account of the SES ADHD relationship in different high income countries.

Our findings are compatible with those reported by Russell et al. [6] in their systematic review of the association of low SES with ADHD in childhood and adolescence. In addition, an association of low SES in early childhood with ADHD in later childhood is reported by other cohort studies [8–17] although different SES measures and methods of analysis limit comparison with our findings. Social gradients by household income in early childhood are reported in three studies [8, 9, 13]. The paper comparing UK and Brazilian cohorts reported an association in both cohorts with hyperactivity measured by the Strengths and Difficulties Questionnaire (SDQ) but not with “any ADHD disorder”, defined as ADHD sub-types (hyperactive-impulsive, inattentive and combined), measured by the Development and Well-Being Assessment (DAWBA) set of questions [10]. Three studies [12, 14, 17] found a social gradient in income trajectories in the early life course with increasing risk from never experiencing low income to persistent low income. Three studies [7, 8, 15] reported a social gradient in ADHD in later childhood by maternal education measured in early childhood. A further study [16] reported an association of ADHD with early childhood SES using a measure of low social class combining low parental education, low occupational status, and low income.

Comparison of findings for countries included in our study are consistent with findings from comparable UK and Swedish cohorts [7, 8, 12–14]. An Australian study using the LSAC K cohort (children enrolled at 4 years of age, versus the LSAC B cohort enrolled at birth) reported a social gradient by maternal education, but not by household income [44]. A paper based on the Quebec Longitudinal Study of Kindergarten Children (QLSKC) [45] reported an association of household income with ADHD measured when the child was 6 years old consistent with our findings from the QLSCD; however, in contrast to QLSCD findings of no association with ICESD level of maternal education measured when the child was 6 months of age, they reported an association with parental education based on whether or not the parent had a high school diploma by the age of 30. A 2007 cross-sectional study based on a nationally representative sample of the US child population, reported no association of ADHD with low maternal education, but a clear association with low income consistent with the US NLSY cohort findings, although reporting a higher prevalence rate of 8% [46]. A Dutch case-control study, nested within a cohort study, found no association of ADHD for low parental education during early childhood [47].

### Interpretation

The findings of this study are broadly consistent with the conclusion of Russell et al.’s systematic review [6] that the association of ADHD with SES is found in different countries. Despite wide variation in ADHD prevalence, major differences in purchasing power parity and income distribution (see Table 3), and in levels of educational attainment (see Table 4), the findings indicate that, in the rich countries studied, high income or high maternal educational status in early childhood is associated with a lower risk of ADHD at 9–11 years. The pathways by which early childhood household income and maternal education exposure exert their influence on risk of ADHD later during childhood are likely to be complex and have not been fully elucidated. Low household income and/or low maternal education may expose children to established socially-related risk factors for ADHD (e.g., inconsistent parenting, family adversity, maternal anxiety/depression), across the early life course. Further, these children who are exposed to low household income and/or low maternal education at a young age are likely to experience longer periods of exposure to risk factors than their more advantaged peers, thereby increasing inequality in ADHD during later childhood. Alternative explanations, such as bidirectional pathways, where the child’s ADHD affects parents’ capacity for earning household income, also have been proposed [48]. However, by including income before ADHD diagnosis in our analyses, we have shown that the direction of association flows from income to ADHD and not the other way around. Furthermore, Russell et al. [7], in a large population-based study, found no evidence of income reduction associated with ADHD over the child’s first 7 years of life. Another alternative explanation that might result in reverse causation is ADHD symptoms in parents leading to poor economic outcomes in adulthood at the same time as increasing the risk of their children developing ADHD. However, both inherited and non-inherited factors play a role in ADHD and their effects are interdependent [49]. The social gradients observed in our EPOCH study are likely to reflect a linear, dose-response relationship to exposure across income and maternal education categories to socially-related risk factors over the early life course in combination with inherited risk. Low maternal education was associated with a higher risk of ADHD in late childhood than low income in the pooled estimates suggesting that maternal education in early childhood might have more influence than household income on ADHD in late childhood. The absence of a gradient in maternal education in the Quebec cohort combined with the higher likelihood in the middle income group may be partly explained by long delays in the public system to access appropriate psychometric ADHD evaluation such that parents of more favorable socioeconomic status (i.e., more educated, higher income) can opt to pay for private services for testing rendering their children more likely to have an ADHD diagnosis [50].

### Strengths and limitations

Harmonization of the SES measures across cohorts is one of the strengths of this study; however, as discussed above, wide variations in income distribution, purchasing power, and distribution of maternal education levels due to social and educational policy differences remain between cohorts. By analysing the effect of household income and maternal education separately, the analysis avoids potential collinearity and allows assessment of whether the effect of these two commonly used SES measures on late childhood ADHD differs. The analysis reduced the likelihood of overcontrolling for SES by excluding socially-related variables which are potential mediators, rather than confounders, of the SES ADHD relationship. All cohorts collected data on household income and maternal education prospectively within the child’s first five years of life, ranging from pregnancy to the fifth year. Non-participation and differential attrition were accounted for by weighting and/or imputation in all cohorts except for Southeast Sweden, which used administrative whole population data rendering weights unnecessary. The longitudinal nature of the analysis combined with the adjustment for differential attrition strengthen the causal interpretation of our estimates.

Several limitations should be taken into account when interpreting findings. Similar to previous worldwide comparisons, prevalence rates of ADHD varied widely [1, 2]. ADHD was based on parental report in all cohorts, except the ABIS cohort. While the use of diagnostic data harmonized across cohorts to address criticisms regarding methodological differences was a study strength, informant bias may have led to overreporting by low SES parents. However, Visser et al. [51] found parent-reported prevalence rates compatible with rates based on administrative data; and, Russell et al. [8] found no evidence of overreporting in disadvantaged groups. Furthermore, others have suggested a socioeconomic gradient exists for ADHD management based on measures of symptom severity and medication use, whereby higher socioeconomic status was associated with superior treatment response and reduction in ADHD symptomatology [52]. Alternatively, financial and insurance limitations on access to diagnostic services in some countries, as discussed above for Quebec, may partially explain variation in prevalence rates as may differences in which professionals are licensed to diagnose in different countries. Teachers are frequently involved in identifying hyperactive children [53] and the extent of their involvement may vary by country. The US prevalence rate is based on a nested question about hyperkineses/hyperactivity/minimal brain dysfunction/ADHD that asked only if the informant identified the child as having a limiting condition; this may have precluded identification of those with inattentive phenotypes or those with ADHD sufficiently managed that did not interfere with daily functioning. Although the pooled estimates for maternal education are consistent with those reported by Russell et al. [6], caution should be employed in interpreting them due to high levels of heterogeneity.

## Conclusions

We have shown that children who were in high income households or had more educated mothers in early childhood were at lower risk of ADHD at age 9–11 in different high-income countries. Pooled estimates indicate that risk of ADHD in later childhood is more strongly related to early childhood exposure to maternal education than household income. We have estimated the percentage difference in ADHD prevalence between the highest and lowest levels of income and maternal education in each cohort population using SII, a measure of absolute inequality. In combination with relative inequality, absolute inequality provides a more complete account of the SES-ADHD relationship in the different cohort populations. The findings of this study confirm the association of early childhood SES with ADHD in later childhood; however, the explanation for this relationship remains unclear. Country-level social, economic, and political decisions have a major influence on household incomes during early childhood and maternal education in mothers’ own childhood suggesting policies that promote adequate levels of household income over the child’s early years and high levels of education, especially among women, have a potential role in reducing the risk of later ADHD and narrowing the social gradient. Health and educational services can contribute by providing support for parents through their children’s early years. Future research should examine the mediators and moderators of the SES-ADHD pathway in early childhood to identify possible interventions to minimise risk associated with low income and low maternal education.

## Supporting information

S1 Appendix Details of data analysis & available data files. (DOCX)

S1 Table Bivariate analysis of ADHD by household income and maternal education and baseline confounders. (CSV)
