# Supplementary material for: Social gradients in ADHD by household income and maternal education exposure during early childhood: Findings from birth cohort studies across six countries
Source: PLoS One. 2022 Mar 16;17(3):e0264709. doi: 10.1371/journal.pone.0264709 (PMC8926184; doi:10.1371/journal.pone.0264709)
Supplement: S1 Appendix — (DOCX) [file pone.0264709.s001.docx]

**S1 Appendix.**

**Details of Data Analyses.**

| **Cohort** | **Software** | **Sampling weights** | **Censoring weights** |
| --- | --- | --- | --- |
| NLSCY | R v 3.6.1 | Yes | Yes |
| GenR | R v 3.6.1 | No | Yes |
| ABIS | Stata v 15 | No | No |
| LSAC | Stata v 15 | Yes | Yes |
| US NLSY | SPSS v 27 | Yes | No |
| MCS | SPSS v 25 | Yes | Yes |
| QLSCD | SPSS v 25 | Yes | Yes |

NLSCY (Canada, all provinces) data were analyzed using R version 3.6.1. Longitudinal weights were applied to adjust both for sampling design and loss to follow up (Statistics Canada. Special Surveys Division. 2006). These weights were estimated by first identifying homogenous groups whereby participants with similar likelihood of response (estimated using logistic regression) are grouped together and sample design weights for respondents are adjusted to account for non-response. Then, post-stratification adjustment is carried out so that the weighted estimates reflect the reference population, in this case all Canadian children eligible for selection into the study at baseline (1994). For our subsample, this means all children aged 0 to 1 years on December 31, 1994.

MCS (UK) data were analyzed using SPSS version 25. Weights used by MCS are described in detail elsewhere (Mostafa 2014). In brief: single and multiple imputation were first used to estimate missing response values. Then, a logit model was applied to estimate probability of non-response using imputed datasets. The predicted probabilities were used to estimate weights that adjust both for attrition and sampling design.

QLSCD (Quebec, Canada) data were analyzed using SPSS version 25. Weights were used to adjust for non-response and to represent the target population of the survey. This was accomplished by; estimating cross-sectional weights for participants in the 2002 wave, excluding children who had passed away and whose families had moved from Quebec and finally, adjusting 2002 weights for non-response in the 2008 survey. Non-response weights were estimated using the score method (Fontaine and Courtemanche 2009)

LSAC (Australia) data were analyzed using Stata version 15. Weights were used to adjust for sampling distribution so that the population of children at follow-up could represent the population originally sampled. In addition, the weights adjusted for non-response at every wave of the survey (Norton and Monahan 2015).

GenR (Rotterdam, The Netherlands) data were analyzed using R version 3.6.1. Generalized linear models with a log link were used with stabilized inverse probability weights to adjust for differential non-response. Generalized estimating equations were used for robust variance estimation using package geepack. Censoring weights were built using the methodology proposed by Hernan and Robins (Hernán and Robins 2020). In brief, the denominator of weights was defined as the probability of not being censored and was estimated using a logistic regression with hypothesized predictors of loss to follow-up. The numerator was the observed probability of exposure which was the probability of belonging to a specific maternal education tertile (for analyses looking at the effect of maternal education) and income tertiles (for analyses looking at the effect of income tertile).

USNLSY (USA) data were analyzed using SPSS version 27. Custom weights were used to adjust for sample attrition of mothers and children and for oversampling and over-representation of minority samples. Due to the complex survey design, the National Longitudinal Survey provides an online custom weighting program (http://nlsinfo.org/weights/childya) used to generate a custom set of survey weights that adjust for both the survey design and for using data across multiple years. Custom weights were obtained for participants included within the analyzed sample (born to USNLSY79 mothers in 1988 to 1996, inclusive).

# **Supporting References.**

Fontaine, Catherine, and Robert Courtemanche. 2009. "Analysis of attrition in the Longitudinal Study of Child Development in Quebec (ÉLDEQ) from 1998 to 2008 ." *Statistics Canada Symposium 2009. Longitudinal Surveys: from Design to Analysis.* Ottawa, Canada: Statistics Canada . 298 - 303.

Hernán, MA, and JM Robins. 2020. *Causal Inference: What If. .* Boca Raton: Chapman & Hall/CRC.

Jaddoe, VW, JP Mackenbach, HA Moll, EA Steegers, H Tiemeier, and FC Verhulst. 2006. "The Generation R Study: Design and Cohort Profile." *European Journal of Epidemiology* 21: 475-84.

Jetté, M, and L Groseilliers. 2000. *Survey description and methodology.* Québec: Institut de la statistique du Québec.

Mostafa, Tarek. 2014. *Millennium Cohort Study: Technical Report on Response in Sweep 5 (Age 11).* London, UK: Centre for Longitudinal Studies.

n.d. *National Longitudinal Survey of Youth, Children and Young Adults 1979.* U.S. Bureau of Labor Statistics. Accessed 05 18, 2020. https://www.nlsinfo.org/.

Norton, A, and K Monahan. 2015. *LSAC Technical paper No. 15. Wave 6 weighting and non-response.* Melbourne, Australia: Australian Bureau of Statistics.

Statistics Canada. Special Surveys Division. 2006. *Microdata User Guide National Longitudinal Survey of Children and Youth Cycle 6, September 2005 - June 2005.* Ottawa: Statistics Canada.

Viechtbauer, W. 2010. "Conducting meta-analyses in R with the metafor package." *Journal of Statistical Software* 36 (3): 1-48.

Zou, Guangyong. 2004. "A Modified Poisson Regression Approach to Prospective Studies with Binary Data." *American Journal of Epidemiology* 159 (7): 702-706.

**S1 Table. Bivariate Analysis of ADHD by Household Income and Maternal Education and Baseline Confounders**

| Variables | MCS  UK  (N = 13354)  RR (95% CI) | ABIS  Sweden  (N= 16365)  RR (95% CI) | QLSCD  Quebec  (N= 1334)  RR (95% CI) | LSAC – B  Australia  (N=3759)  RR (95% CI) | GenR  Rotterdam  (N=5100)  RR (95% CI) | US NLSY  USA  (N=3657)  RR (95% CI) | NLSCY  Canada  (N=1356)  RR (95% CI) |
| --- | --- | --- | --- | --- | --- | --- | --- |
| Income at Baseline  High (Reference)  Middle  Low | Reference  1.51 (0.85, 2.68)  3.87 (2.24, 6.68) | Reference  1.26 (0.92, 1.73)  1.90 (1.42, 2.53) | Reference  1.82 (1.09, 3.06)  1.60 (0.93, 2.75) | Reference  0.97 (0.61, 1.53)  1.26(0.81, 1.95) | Reference  1.96 (1.54, 2.50)  1.63(1.26, 2.11) | Reference  1.38 (0.79, 2.42)  1.32 (0.75, 2.33) | Reference  0.53 (0.24, 1.21)  1.93 (0.92, 4.05) |
| Mother Education at Baseline  High (Reference)  Medium  Low | Reference  1.51 (0.90, 2.53)  3.76 (2.15, 6.60) | Reference  2.04 (1.47, 2.81)  4.63 (3.16, 6.79) | Reference  0.84 (0.52, 1.38)  0.88 (0.52, 1.49) | Reference  1.38 (0.91, 2.09)  2.77 (1.70, 4.50) | Reference  2.00 (1.57, 2.55)  2.27 (1.68, 3.06) | Reference  1.08 (.67, 1.73)  1.18 (.60, 2.30) | Reference  1.23 (0.52, 2.92)  1.86 (0.69, 5.06) |
| Child Sex  Girl  Boy | Reference  4.12 (2.65, 6.41) | Reference  3.39 (2.56, 4.48) | Reference  0.98 (0.65, 1.48) | Reference  2.79 (1.82, 4.27) | Reference  1.86 (1.54, 2.24) | Reference  3.69 (2.16, 6.31) | Reference  8.11 (3.50, 18.76 |
| Mother Ethnicity^a^  Ethnic Majority/Born in Country (Ref)  Ethnic Minority / Born outside country | Reference  0.52 (0.28, 0.95) | Reference  1.02 (0.64, 1.61) | Reference  0.95 (0.51, 1.78) | Reference  0.69 (0.45, 1.06) | Reference  1.07 (0.89, 1.29) | Reference  1.39 (0.93, 2.06) | N/A |
| Multiple Births^a^  No (Reference)  Yes | Reference  2.52 (1.06, 5.95) | Reference  2.15 (1.27, 3.65) | N/A | Reference  1.05 (0.40, 2.75) | Reference  1.61 (0.84, 3.09) | Reference  0.40 (0.08, 1.62) | N/A |
| Mother Age at Child Birth^b^  <20 yrs  20-29 yrs  30-39 yrs (Reference)  40+ yrs | 2.44 (1.13, 5.25)  1.37 (0.91, 2.06)  Reference  1.73 (0.56, 5.34) | 5.83 (3.53, 9.63)  1.62 (1.26, 2.09)  Reference  0.53 (0.13, 2.14) | 0.71 (0.17, 3.06)  1.00 (0.66, 1.52)  Reference  0.42 (0.06, 3.15) | 1.91 (0.71, 5.19)  1.42 (0.96, 2.08)  Reference  1.26 (0.53, 3.03) | 2.52 (1.24, 5.13)  1.28 (1.05, 1.56)  Reference  1.26 (0.71, 2.23) | N/A  1.02 (0.68, 1.56)  Reference  N/A | ^15-24 yrs^ 0.10 (0.06, 0.19)  ^25-29 yrs^0.30 (0.13, 0.66)  ^30+ yrs^ Reference  N/A |

Note. ^a^Multiple birth and maternal ethnicity not available (N/A) for select cohorts.

^b^Maternal age categories consolidated due to small n per cell in unweighted tables and reporting restrictions for select cohorts.

**Syntax Code: ADHD SII Calculation**

### set your working directory here: enter the path to the folder where you saved the data file

setwd("~/Concordia/EPOCH/ADHD/Data")

### load the ggplot package after installing it

library(ggplot2)

### import data

df = read.csv('ADHD_prevalences_SII.csv')

cohorts = unique(df$cohort)

############# MATERNAL EDUCATION

### keep rows with prevalence by maternal education category only

dfe = subset(df, exposure == 'education')

### calculate the sum of all the category proportions (will be < 1 if there is missing data)

sumw= data.frame(sum = tapply(dfe$prop, dfe$cohort, sum),

cohort = c('ABIS', 'GenR', 'LSAC', 'MCS', 'NLSCY', 'QLSCD', 'USNLSY' ))

### add this sum back to the data.frame

dfe = merge(dfe, sumw, by = 'cohort', all.x = T)

### calculate a new proportion but of non-missing data

dfe$wc = dfe$prop / dfe$sum

### calculate the number of children per category

dfe$numtert = dfe$wc*dfe$totn

### calculate mid-point of each category

dfe$cfmid = dfe$wc/2

### calculate riddit score of each category

dfe$cf = NA

for (i in 1:length(cohorts)){

dfe$cf[dfe$category == 'low' & dfe$cohort == cohorts[i]] = dfe$cfmid[dfe$category == 'low'& dfe$cohort == cohorts[i]]

dfe$cf[dfe$category == 'mid'& dfe$cohort == cohorts[i]] = dfe$wc[dfe$category == 'low'& dfe$cohort == cohorts[i]] + dfe$cfmid[dfe$category == 'mid'& dfe$cohort == cohorts[i]]

dfe$cf[dfe$category == 'hi'& dfe$cohort == cohorts[i]] = dfe$wc[dfe$category == 'low'& dfe$cohort == cohorts[i]] + dfe$wc[dfe$category == 'mid'& dfe$cohort == cohorts[i]] + dfe$cfmid[dfe$category == 'hi'& dfe$cohort == cohorts[i]]

}

### calculate SII + confidence interval for each cohort using a weighted linear regression

SIIsw = data.frame(cohort = as.character(cohorts),

sii = round(as.numeric(unlist(lapply(cohorts, function(x)

coefficients(lm(prev~cf, data = dfe[dfe$cohort == x,], weights = numtert))[2]))),2),

se =

round(as.numeric(unlist(

lapply(cohorts, function(x)

sqrt(diag(vcov(lm(prev~cf, data = dfe[dfe$cohort == x,], weights = numtert))))[2]

))),2))

### export SIIs and save them in a .csv file in the folder you set as your working directory

write.csv(SIIsw, 'SII_ADHD_EDUCATION_weights.csv', row.names = F)

### Draw plot & save as jpeg file in your working directory

# Define colour palette that is colour blind friendly and has no bright yellow

cbPalettec <- c("#999999", "#E69F00", "#56B4E9", "#009E73","#000000", "#0072B2", "#D55E00", "#CC79A7")

jpeg(filename="SIIw_ADHD_Education.jpeg", width = 3000, height = 2500, res=500)

ggplot(data = dfe,aes(x = cf, y = prev, color = cohort)) +

xlab('Cumulative fraction of population ranked by maternal education') +

ylab('Prevalence of ADHD in maternal education category') +

xlim(0,1) + ylim(0,10) +

geom_smooth(method = "lm", mapping = aes(weight = numtert), se = FALSE,fullrange=T) +

geom_text(aes(x = 0.4, y = 9.4, label = -1.37 ,color = 'QLSCD'), size = 2.5) +

geom_text(aes(x = 0.4, y = 2.6, label = '-0.50',color = "GenR"), size = 2.5) +

geom_text(aes(x = 0.4, y = 4.5, label = -0.79,color = "NLSCY"), size = 2.5) +

geom_text(aes(x = 0.4, y = 3.98, label = -4.43,color = "LSAC"), size = 2.5) +

geom_text(aes(x = 0.4, y = 2.25, label = -3.05,color = "ABIS"), size = 2.5) +

geom_text(aes(x = 0.4, y = 1.4, label = -2.54,color = "MCS"), size = 2.5) +

geom_text(aes(x = 0.4, y = 3.65, label = '-0.74',color = "USNLSY"), size = 2.5) +

scale_colour_manual(values=cbPalettec)

dev.off()

#### delete unwanted files

rm(dfe, SIIsw, sumw, i)

############# Household Income

### keep rows with prevalence by household income tertile only

dfe = subset(df, exposure == 'income')

### calculate the sum of all the category proportions (will be < 1 if there is missing data)

sumw= data.frame(sum = tapply(dfe$prop, dfe$cohort, sum),

cohort = c('ABIS', 'GenR', 'LSAC', 'MCS', 'NLSCY', 'QLSCD', 'USNLSY' ))

### add this sum back to the data.frame

dfe = merge(dfe, sumw, by = 'cohort', all.x = T)

### calculate a new proportion but of non-missing data

dfe$wc = dfe$prop / dfe$sum

### calculate the number of children per category

dfe$numtert = dfe$wc*dfe$totn

### calculate mid-point of each category

dfe$cfmid = dfe$wc/2

### calculate riddit score of each category

dfe$cf = NA

for (i in 1:length(cohorts)){

dfe$cf[dfe$category == 'low' & dfe$cohort == cohorts[i]] = dfe$cfmid[dfe$category == 'low'& dfe$cohort == cohorts[i]]

dfe$cf[dfe$category == 'mid'& dfe$cohort == cohorts[i]] = dfe$wc[dfe$category == 'low'& dfe$cohort == cohorts[i]] + dfe$cfmid[dfe$category == 'mid'& dfe$cohort == cohorts[i]]

dfe$cf[dfe$category == 'hi'& dfe$cohort == cohorts[i]] = dfe$wc[dfe$category == 'low'& dfe$cohort == cohorts[i]] + dfe$wc[dfe$category == 'mid'& dfe$cohort == cohorts[i]] + dfe$cfmid[dfe$category == 'hi'& dfe$cohort == cohorts[i]]

}

### calculate SII + confidence interval for each cohort using a weighted linear regression

SIIsw = data.frame(cohort = as.character(cohorts),

sii = round(as.numeric(unlist(lapply(cohorts, function(x)

coefficients(lm(prev~cf, data = dfe[dfe$cohort == x,], weights = numtert))[2]))),2),

se =

round(as.numeric(unlist(

lapply(cohorts, function(x)

sqrt(diag(vcov(lm(prev~cf, data = dfe[dfe$cohort == x,], weights = numtert))))[2]

))),2))

### export SIIs and save them in a .csv file in the folder you set as your working directory

write.csv(SIIsw, 'SII_ADHD_INCOME_weights.csv', row.names = F)

### Draw plot & save as jpeg file in your working directory

jpeg(filename="SII_ADHD_Income.jpeg", width = 3000, height = 2500, res=500)

ggplot(data = dfe,aes(x = cf, y = prev, color = cohort)) +

#geom_smooth(method = 'lm', se = FALSE) +

xlab('Cumulative fraction of population ranked by income') +

ylab('Prevalence of ADHD in income tertile') +

xlim(0,1) +

geom_smooth(method = "lm", mapping = aes(weight = numtert), se = FALSE,fullrange=T) +

geom_text(aes(x = 0.5, y = 8.2, label = '-6.40' , color = 'QLSCD'), size = 2.5) +

geom_text(aes(x = 0.5, y = 2.5, label = -1.45, color = "GenR"), size = 2.5) +

geom_text(aes(x = 0.5, y = 5.4, label = -5.19, color = "NLSCY"), size = 2.5) +

geom_text(aes(x = 0.5, y = 4.3, label = -1.32, color = "LSAC"), size = 2.5) +

geom_text(aes(x = 0.5, y = 2, label = -1.65, color = "ABIS"), size = 2.5) +

geom_text(aes(x = 0.5, y = 1.1, label = -2.77, color = "MCS"), size = 2.5) +

geom_text(aes(x = 0.5, y = 3.4, label = -1.71, color = "USNLSY"), size = 2.5) +

scale_colour_manual(values=cbPalettec)

dev.off()

**Dataset Metadata: Readme File**

Data used to calculate SII:

‘ADHD_prevalences_SII.csv’

This file contains the following columns:

cohort = Acronym of cohort study

exposure = Indicates early childhood SES measure as either education (maternal education) or income (total household income)

prev = ADHD prevalence % at age 10/11 (for mean age of ADHD diagnosis please see Table 2 in manuscript)

prop = Proportion of category (number of children in category / total number of children at follow-up)

category = Category of maternal education or household income in early childhood as defined in manuscript

totn = total number of children at follow-up

Code used to calculate SII:

ADHD_SII_Calculation.R

Software needed to run code:

R version 4.1.0 (2021-05-18) -- "Camp Pontanezen"

Copyright (C) 2021 The R Foundation for Statistical Computing

Platform: x86_64-apple-darwin17.0 (64-bit)

Studio version Version 1.3.1093

Package ggplot
